# Characteristics of Primary Care Trusts in financial deficit and surplus – a comparative study in the English NHS

**DOI:** 10.1186/1472-6963-6-64

**Published:** 2006-06-01

**Authors:** Padmanabhan Badrinath, Rosemary Anne Currell, Peter M Bradley

**Affiliations:** 1 Suffolk West Primary Care Trust & University of Cambridge, Thingoe House, Cotton Lane, Bury St Edmunds, Suffolk, UK; 2 Suffolk West Primary Care Trust, Thingoe House, Cotton Lane, Bury St Edmunds, Suffolk, UK; 3 Suffolk West Primary Care Trust & University of East Anglia, Thingoe House, Cotton Lane, Bury St Edmunds, Suffolk, UK

## Abstract

**Background:**

Recently the financial status of primary care trusts has come under considerable scrutiny by the government, and financial deficits have been blamed on poor local management of resources. This paper examines the factors that differ between those Primary Care Trusts (PCT) in financial deficit and those in surplus, using readily available data at PCT level. PCTs are the National Health Service organisations in England responsible for improving the health of their population, developing primary and community health services, and commissioning secondary care services.

**Methods:**

A descriptive comparative study using data from 58 PCTs; 29 in greatest financial surplus and 29 in greatest deficit in the English National Health Service.

**Results:**

Nearly half the study deficit PCTs (14 out of 29) are in the East of England and of the 29 surplus PCTs, five each are in Birmingham and Black Country Strategic Health Authority (SHA), and Greater Manchester SHA. The median population density of the deficit PCTs is almost seven times lower than that of surplus PCTs (p = 0.004). Surplus PCTs predominantly serve deprived communities. Nearly half the surplus PCTs are 'spearhead' PCTs compared to only one of the deficit PCTs. Percentage population increase by local authority of the PCT showed that on average deficit PCTs had 2.7 times higher change during 1982–2002 (13.37% for deficit and 4.94% for surplus PCTs). Work pressure felt by staff is significantly higher in deficit PCTs, and they also reported working higher amount of extra hours due to work pressures. The proportion of dispensing general practitioners is significantly higher in deficit PCTs 40.5% vs. 12.9% (p = 0.002). Deficit PCTs on average received £123 less per head of registered population compared to surplus PCTs.

**Conclusion:**

The two groups of PCTs serve two distinct populations with marked differences between the two. Deficit PCTs tend to be in relatively affluent and rural areas. Poor management alone is unlikely to be the cause of deficits, and potential reasons for deficits including rurality and increased demand for health services in more affluent communities need further in-depth studies.

## Background

The financial status of many NHS organisations including Primary Care Trusts (PCTs) has come under considerable scrutiny in recent months and this has attracted much political and media interest. Department of Health figures show that in the year 2004–05, 90 of the 303 PCTs in England were overspent, 92 broke even and 121 were in surplus [1]. PCT deficits ranged from 0.1% to 6.7% of their annual revenue resource limit, and surpluses ranged from 0.1% to 2.6%. As a whole, the PCTs were overspent by a total of £271 million and this was predicted to rise to over £300 million [2]. The Government refers to the extra investment it has made in the NHS in recent years and is insistent that Primary Care Trusts and hospitals have to balance their books, pay back any overspend and will not be given any extra money to cover deficits [3]. It is also a statutory obligation for all NHS organisations to reach financial balance at the end of the financial year. According to the think tank "Reform" [4] "the government is handling the financial problems as a series of local deficits which are the results of faults by local management".

However, the King's Fund found in their study [5] of hospital finances across the NHS that "hospitals' financial problems are not always the result of inefficiency or poor management and there are other factors including stranded costs, imperfections in the design of payment by results tariff and the "legacy costs" – the legacy of past investment and service delivery which cannot readily be reversed". Similarly it is also plausible that multiple factors could be affecting the financial status of PCTs, including how NHS resources are allocated, their geographical and demographic nature, how services are configured, demand for services, as well as the management ability of these organisations.

It is particularly important for PCTs trying to cope with a deficit to understand possible contributory factors. As a first step, this paper examines the factors that differ between those PCTs in financial deficit and those in surplus, using PCT level data from various sources.

## Methods

Our aim was to determine the factors that might influence the financial status of PCTs in England. A wide range of factors might impact on their financial status and a number of key areas were identified in advance of data analysis. Only variables considered relevant to the research question, with few missing values, and showing discrimination between PCTs were included. These were measures of: PCT demographic and socioeconomic profile, health and health needs, performance and staffing profile, primary and secondary care activity, and resource allocation.

### Study sample

Data on the financial status of the PCTs was gathered from the DH web site [1]. The financial deficit as a % of annual turnover was arranged in descending order with deficit PCTs ranked highest and surplus PCTs ranked lowest. Just under 10% of the PCTs from both the top and the bottom of this list were selected, which provided the two study groups with 29 PCTs in each. Information on the 58 selected PCTs was then drawn from a variety of sources. Due to this triangulation of data sources, not all data sets refer to the same years. For example when we calculated resource allocation per head of population, the resource revenue limit was for 2004–05 and the denominator populations were for 2003 in the case of resident and 2001 in the case of registered population.

#### 1. PCT profile

The variables included in the analysis are demographic, morbidity & mortality, geographic, socio-economic, performance and staffing. This information was downloaded from the NHS National workforce projects web page [6,7]. We also ascertained how many of the PCTs in the study were spearhead PCTs. There are 88 spearhead PCTs in England which were identified by the government [8] using information on deprivation, mortality from cancer and heart disease as well as life expectancy, to determine the areas that face the greatest health challenges.

#### 2. Primary care

QOF achievement information at PCT level for the year 2004–05 was downloaded from the Health and Social Care Information Centre [9]. GMS practice activity data for the year 2003 (the latest year available) was abstracted from the National Primary Care Database and this included information on number of practices, GPs and dispensing GPs at PCT level [10].

#### 3. Secondary care

Finished consultant episodes at PCT level was retrieved from the Health Care Workforce PCT profile [7].

#### 4. Resource allocation

PCTs receive their financial allocation from the DH on the basis of a weighted capitation formula, which takes into account factors including age and gender structure of the population, geographic and social factors, and morbidity and mortality rates. Closing composite distance from target for the year 2004–05 and 2006–07 for all the PCTs was abstracted from the Unified Exposition Books [11,12]. Exposition books provide information on resource allocation and the basis for their calculations. The allocation to each PCT takes account of a general policy of moving the PCT's actual financial position towards its target financial position, calculated on the basis of its weighted population. This is known as the 'distance from target' (DFT).

### Analysis

Data for the 58 PCTs were collated and analysed using the Statistical Package for Social Sciences version 14 [13]. The two groups of PCTs – those in deficit and surplus – were compared on different variables using Chi^2 ^or Fisher's exact test for categorical variables, and 't' test or rank test for continuous variables as appropriate. Due to the multiple testing of many variables, a p value of <0.01 was considered statistically significant.

## Results

Nearly half of the deficit PCTs (14 out of 29) are in the East of England and one third (9 out of 29) are concentrated in the Norfolk Suffolk and Cambridgeshire Strategic Health Authority (SHA). Of the 29 surplus PCTs 5 each are in Birmingham and Black Country SHA, and Greater Manchester SHA.

### PCT profile

There was no difference between the two groups of PCTs in mean age or proportion of those aged under 6, or 65 years and over. Fifteen of the 29 surplus PCTs belong to the Office for National Statistics (ONS) bands 1 to 4 as opposed to 3 deficit PCTs (Fisher's exact test p = 0.000); (1 – inner city, 2 – Port or mining industrial towns, 3 – mixed economy urban centres, 4 – Service, education, resort and retirement areas). Deficit PCTs predominantly belong to band 5 and 6 (5 – Prosperous and growth centres, 6 – Mixed urban, rural or coast locations). There is marked difference in the population density of the two groups of PCTs, with the median density being nearly 7 times lower in the deficit PCTs (p = 0.004). Nearly half (14) surplus PCTs were spearhead PCTs compared to only one of the deficit PCTs.

Table 1 shows the resident and registered populations, and resource allocation per capita for the two groups of PCTs. Percentage population growth for the UK by local authority of the PCTs show that for the years 1982–2002, on average, deficit PCTs had 2.7 times higher change (13.37% for deficit and 4.94% for surplus PCTs). Table 2 shows how the socioeconomic, health and health needs variables of the two groups of PCTs differ significantly.

There were no major differences in the performance of the two groups of PCTs using the Health Care Commission's Star rating data [7] apart from access to services for early unintended pregnancy in which the deficit PCTs fared better (p = 0.003). In the overall star rating analysis 5 out of 29 deficit PCTs had no star as opposed to none of the surplus PCTs which was not statistically significant (Fisher exact, two sided p = 0.052).

There were no significant differences in staffing variables, except that surplus PCTs employed 1 Health Visitor (HV) per 5137 registered population as opposed to 1 HV per 6570 by deficit PCTs (p = 0.002). The work pressure felt by staff is significantly higher (p = 0.006) in deficit PCTs (3.3) compared to surplus PCTs (3.2) and a higher % of staff in deficit PCTs (66.3% vs. 62.0%) also reported working more extra hours owing to work pressures; (p = 0.005).

### Primary care

The average practice size (PCT population divided by number of practices) was significantly higher in deficit PCTs (7132) compared to surplus PCTs (5555) (p = 0.000). The proportion of dispensing GPs was significantly higher in deficit PCTs 40.5% vs. 12.9% (p = 0.002). Practices in deficit PCTs had a marginally higher average total QOF points but this was not statistically significant (987.7 vs.946.6, p = 0.035).

### Secondary care

On average deficit PCTs had a higher number of finished consultant episodes (FCE) per 1000 population (3.57/1000 population for deficit and 3.09/1000 for surplus PCTs, p = 0.003).

### Resource allocation

Deficit PCTs received on average £205 per head of resident population and £123 per head of registered population less than the surplus PCTs (p = 0.000). Comparison of 2004–05 closing composite DFT showed a marked difference, with deficit PCTs being over target by an average of 1.5% and surplus PCTs under target by 2.73% (p = 0.001). However in their 2006–07 allocation deficit PCTs were under target on average by 1% in 2006–07.

### Ethical approval

Not required.

## Discussion

The factors influencing financial status of PCTs are probably very complex and have not been studied in detail. It is unlikely that deficits are due to poor management alone, and if this were the case, one would not expect to see a clustering of the PCTs most in deficit in one region of the country as observed in this study. The aim of this study was to identify factors that differ between PCTs in financial deficit and those in surplus, and a number of striking differences were observed.

The analysis compared the 29 English PCTs most in deficit with the 29 PCTs in the best financial position. The profiles of these PCTs showed that the two groups serve quite different populations. The PCTs in financial surplus have been shown to be in the less prosperous areas of the country, with significantly higher levels of deprivation on measures such as unemployment, educational achievement, proportion of benefit claimants, proportion of poorer housing, standardised mortality ratios, and life expectancy for men. These are the factors that are taken into account in the distribution of resources within the NHS, with positive discrimination in favour of deprived areas, intended to take account of their extra health care needs and to reduce health inequalities.

As other authors have noted: "The current resource allocation formula responds well to the higher relative needs of the urban populations. Yet, it is generally agreed that the NHS (and particularly hospital services which account for the greater proportion of NHS expenditure) has relatively little to contribute towards the reduction of health inequalities compared to other sources of variation such as income distribution, education and so on. Thus, the targeting of additional services at urban deprived populations is likely to be an ineffective response to health inequalities. It is one, moreover, that introduces a new form of inequity by underestimating the needs of rural populations" [14].

Other government initiatives such as the introduction of health trainers, which are being targeted in the first instance at deprived communities [15] and which are being funded outside PCT allocations are to be welcomed as they are more likely to tackle some of the root causes of health inequalities.

The results of the study further demonstrate the effects of Resource Allocation when the differences in funding per head of population in the two groups of PCTs are considered. It is noteworthy that deficit PCTs receive on average £205 less per resident population and £123 less per registered population than the surplus PCTs (Table 1). It is also interesting to note that deficit PCTs which were over target in the 2003–04 allocation cycle have moved to become under target in the 2006–07 cycle. This big swing away from target reflects the components of the resource allocation formula, especially its sensitivity to changes in population size. In the inter census years this depends on population projections, which in growth areas such as those of deficit PCTs are harder to predict accurately the further we move away from the census year, thus creating a possible mismatch between population size and resource allocation. Demonstrating this with empirical data would be of value but this was not a part of our exploratory study.

The study has also shown other population differences between the two groups of PCTs which may be independently affecting their financial status. Both the mean resident and registered populations of the deficit PCTs are higher than those of the surplus PCTs. Moreover, the populations of deficit PCTs have grown at a much faster rate than the surplus PCTs in the last two decades. National Statistics [16] show that there have been big variations in the English regions, with the North East and North West regions experiencing a decline in population while the South West, East and South East have seen population growth of 10 per cent or more. Although population changes are part of the resource allocation formula it is possible that it is not sensitive enough to take account of this growth. In the 2006–07 allocation, Office of the Deputy Prime Minister growth area adjustment has been included for the first time to account for this increase.

The average practice size differed between the PCTs with surplus PCTs having a smaller list per practice, which could be due to the greater number of single handed practices in inner cities. This may affect primary care availability and utilisation which could be lower in single handed practices. Rural areas tend to invest more heavily in primary care and are likely to provide a wider range of services [17]. There is also a significant difference between the two groups in the proportion of dispensing GPs, which are three times higher in the deficit PCTs. This is probably accounted for by the rural nature of many of the deficit PCT. The costs to the PCTs of dispensing practices are much higher than for non-dispensing practices.

To our knowledge rurality is not explicitly included in the current resource allocation formula in England, although a rurality weighting is applied for calculating general medical services payment [18] and the Department of Health uses a rurality index in emergency ambulance cost adjustment. In a Commons Hansard Written Answers in 2001, the Minister stated, "Earlier studies have not identified evidence of need for health care associated with rurality that is not already covered within the formula. However some services cost more to provide in rural areas. An emergency ambulance cost adjustment has been included in the formula since 1998–99" [19]. Nevertheless "Scotland, Wales and Northern Ireland all operate funding formulae which include a specific allowance for rurality" [20].

Asthana and Gibson [21] recently demonstrated that the pattern of PCT financial deficits "implies that NHS funding provides insufficient resources for rural areas, for comparatively affluent areas, and, most particularly, for areas that are both rural and affluent. Traditional measures of poverty are not applicable to rural areas as often rural poverty is hidden" [17]. However introduction of the Index of Multiple Deprivation 2000 and 2004 [22] increases the possibility of identifying rural communities with greater health and social needs.

It could be expected that there might be some difference in the performance of the PCTs in the different groups, but the analysis showed no major differences between the two groups of PCTs, on the Healthcare Commission Star Rating measures, except variation in financial management [23]. This suggests that in spite of resource constraints, PCTs are performing similarly in both groups. Other performance measures considered in this study showed that deficit PCTs had a higher FCE per head of population than the surplus PCTs, which might reflect increased demand from informed consumers of healthcare, or differences in GP referral patterns.

It is possible that supplier induced demand is playing a role in the increased activity seen in deficit PCTs, but data to substantiate this is not readily available and needs further exploration. The recent White Paper [24] has emphasised the desired shift from secondary to primary and community care, and stated "Unless this White Paper strategy is pursued and the consequent service reconfiguration takes place – some local financial imbalances may never be corrected", demonstrating that even where local financial mismanagement may exist, it alone cannot account for the current financial status of deficit PCTs.

The study points to a positive association between deprivation and financial surplus, for example 14 of the 29 surplus are spearhead PCTs. Whilst the principle of targeting funding at areas of greatest need is not questioned here, it should be recognised that these areas also have access to funding sources to address the wider determinants of health that are not available to more affluent communities. According to Heart of Birmingham PCT [25], which is both a spearhead PCT and is the PCT with the maximum surplus "Our budget is around £360 million a year. As a "Spearhead PCT" our income is expected to grow even more over the next three years so that we can work with other organisations, particularly the city council and voluntary sector to give people more opportunity to live healthier lives".

Some of the limitations of this study have to be borne in mind while interpreting the results. This study only included PCTs at both extremes of the financial status spectrum (just under 10% of each). Different data sources covering different time periods were used, and although this could be a weakness, as the data were gathered for purposes other than the one under study (financial status), and came from multiple sources, this further strengthens the study observations. Qualitative information from clinicians, managers and patients from the two groups of PCTs might have thrown further light on the difference. We did not attempt to gather this data due to resource constraints. Another important point to note is that associations observed do not imply causation. Finally, as the analysis was undertaken at group level the issue of ecological fallacy needs to be borne in mind. In other words, apparent associations measured at a group level (i.e. PCTs in surplus or deficit), may not necessarily be applicable to individual PCTs.

## Conclusion

This preliminary study has demonstrated that the PCTs in England that are most in financial deficit represent a markedly different population from the PCTs most in surplus. The PCTs in financial surplus appear to be those that have benefited from the positive discrimination of the Department of Health's Resource Allocation Formula. With the Government committed to reducing health inequalities in the UK, targeting funding to those areas of the country with the poorest health must be a sound policy objective. Indeed a recent study [26] on inequalities in access to medical care by income, found that in the UK, "pro rich inequity" in access to specialist care seen in 1996 had already been reversed in 2000, with the poor having equal access to specialist care. However, our study raises two issues for policy consideration. The first is to question whether the resource allocation formula in its current form takes adequate account of rural poverty and the particular problems of providing health services to widely dispersed communities. The second issue is the well recognised problem of how to narrow the gap in national health inequalities and yet at the same time continue to improve the health of the population as a whole, and also provide health services of increasing quality and complexity. It would be helpful to the PCTs coping with financial deficit if this difference in funding levels was more widely acknowledged, together with the particular difficulties for health service managers of providing services to rapidly growing and changing populations in areas that have inherited the problems of essentially rural, scattered communities. We recommend that the Department of Health conducts a detailed study of the factors contributing to the financial status of PCTs and in particular to test the following hypotheses; costs of providing health services in rural areas are higher than in urban areas independent of the population's health status; the demand for health services in affluent communities and thus health care expenditure is higher than the demands in more deprived communities.

## Competing interests

The author(s) of this paper are all employed by a PCT, which is in financial deficit.

## Authors' contributions

PB developed the original idea, identified the data bases and abstracted the data, performed the analysis and wrote the manuscript. RAC abstracted the data, performed the analysis, interpreted the results and wrote the manuscript. PMB was involved in the further development of the original idea, interpreted the results and wrote the manuscript. All authors approved the final version of the manuscript. PB and RAC are the guarantors of the paper.

## Pre-publication history

The pre-publication history for this paper can be accessed here:



## Figures and Tables

**Table 1:** Population & resource allocation in 2004–05 by PCT status

Status	Mean resident population*	Mean registered population^†^	£ per resident population^‡^	£ per registered population^‡^
Deficit	150337	152658	1061	1046
Surplus	142547	151422	1266	1169

* In 2003 † In 2001 ‡ The differences are statistically significant (p = 0.000)

**Table 2:** Socio economic, health and health care characteristics by PCT status

Characteristic* (Mean)	**Status**		**p value**
	
	**Deficit**	**Surplus**	
Long term sickness benefit claimants %	4.1	6.7	0.000
Disability allowance claimants %	5.3	7.4	0.000
Lone parents with dependant children %	5.0	7.2	0.000
Home care hours each week per capita	0.06	0.04	0.008
People without GCSEs %	26.8	33.9	0.000
Income support & JSA claimants %	2.5	3.9	0.000
Least valuable housing %	14.6	39.4	0.000
Pensioners living alone %	14.3	15.4	0.03^†^
% Homeless families as a % of all homeless families in England	0.22	0.75	0.02^†^
Standardised mortality ratio (SMR)	92.7	104.0	0.000
Male life expectancy in years	76.3	74.3	0.000
Female life expectancy ratio	81.7	80.6	0.37^†^

* Not all data is available for all PCTs † Statistically not significant
